# *LaLiga* Lockdown: Conditioning Strategy and Adaptation to In-Game Regulations during COVID-19 Pandemic Prevented an Increase in Injury Incidence

**DOI:** 10.3390/ijerph19052920

**Published:** 2022-03-02

**Authors:** Víctor Moreno-Pérez, Jon Patricios, Narciso Amigo de Bonet, Miguel Ángel Buil, Josu Díaz de Alda, Andrés Fernández-Posada, Oliver Gonzalo-Skok, Sergio Jiménez-Rubio, Alberto Lam, Josean Lekue, Roberto López-Del Campo, Alejandro López-Valenciano, Gil Rodas, José Romero-Sangüesa, Xabier Valencia-Murua, Xavier Yanguas-Leyes, José Conde, Juan Del Coso

**Affiliations:** 1 Sports Research Center, Miguel Hernandez University of Elche, 03202 Alicante, Spain; vmoreno@umh.es; 2 Center for Translational Research in Physiotherapy, Department of Pathology and Surgery, Miguel Hernandez University of Elche, 03550 San Joan, Spain; 3 Wits Sport and Health (WiSH), Faculty of Health Sciences, School of Clinical Medicine, University of the Witwatersrand, Johannesburg 2193, South Africa; jpat@mweb.co.za; 4 Medical Department, Real Club Deportivo Espanyol, 08940 Barcelona, Spain; narciso.amigo@rcdespanyol.com; 5 Department of Sports Medicine, Levante U.D., 46360 Valencia, Spain; miguel.buil@ivre.es; 6 Department of Sports Medicine, IVRE-Institut Valencià de Recuperació Esportiva, 46010 Valencia, Spain; 7 Medical Service Department, Alavés Sociedad Anónima Deportiva, 1007 Vitoria-Gasteiz, Spain; iosuposu@hotmail.com; 8 Club Athlético Osasuna Medical Service, 31192 Pamplona, Spain; andresfernandez@osasuna.es; 9 Faculty of Health Sciences, University of San Jorge, 50830 Zaragoza, Spain; oligons@hotmail.com; 10 Performance Department, Getafe C.F., 28903 Madrid, Spain; sjimenezrubio@yahoo.es; 11 Department of Sports Medicine, Leganes C.D., 28918 Madrid, Spain; serviciosmedicos@cdleganes.com; 12 Medical Services, Athletic Club, 48196 Lezama, Spain; j.lekue@fundacionathleticclub.eus; 13 Department of Competitions and Mediacoach, LaLiga, 28043 Madrid, Spain; rlopez@laliga.es; 14 Centre for Sport Studies, Rey Juan Carlos University, 28943 Fuenlabrada, Spain; alejandro.valenciano@urjc.es; 15 Futbol Club Barcelona, Medical Department, FIFA Center of Excellence, 08028 Barcelona, Spain; gil.rodas@fcbarcelona.cat (G.R.); xavier.yanguas@fcbarcelona.cat (X.Y.-L.); 16 Sports Performance Department, Alavés Sociedad Anónima Deportiva, 01007 Vitoria-Gasteiz, Spain; joseromeropf@hotmail.com; 17 Medical Services, Sociedad Deportiva Eibar, 20600 Eibar, Spain; xabi.valencia@sdeibar.com; 18 Performance Department, Sevilla C.F., 41005 Sevilla, Spain; joseconde@ugr.es

**Keywords:** soccer, injury risk, elite athlete, football performance, muscle injury

## Abstract

The first division of Spanish professional football (*LaLiga*) was suspended for 12 weeks as part of the policies enforced by health authorities during the first wave of COVID-19. During this period, players were confined to home for 8 weeks, followed by a club-based retraining period of 4 weeks. Afterwards, *LaLiga’s* teams completed 11 matches, with approximately 3 days of recovery between matches, to finish the competition. The aim of this investigation was to determine whether there is a difference in mean injury incidence in *LaLiga* players between the pre-lockdown period and post-lockdown period. A total of 277 players belonging to 11 teams competing in *LaLiga* were monitored during the 2019–2020 season. Injury incidence in the 27 matchdays completed before the lockdown was compared to the last 11 matchdays completed after the resumption of the competition. In comparison to the period before the suspension, the resumption of the championship did not significantly affect the injury incidence (4.2 vs. 5.4 injuries per 1000 h of exposure, *p* = 0.338). Injury incidence before suspension and after resumption of the competition was similar for muscle (2.6 vs. 3.4 injuries per 1000 h of exposure, *p* = 0.152) and ligament injuries (0.8 vs. 0.4 injuries per 1000 h of exposure, *p* = 0.062). The resumption of the competition also did not modify the distribution of injury according to body location (*p =* 0.948), injury type (*p* = 0.766), mode of onset (*p* = 0.614), severity (*p* = 0.065), or player position (*p* = 0.295). In summary, mean injury incidence in *LaLiga* players was similar before and after the lockdown. It is probable that the conditioning strategy adopted by clubs before the resumption of *LaLiga* and the adaptation of some in-game regulations helped to avoid an increased injury rate after the lockdown.

## 1. Introduction

Coronavirus disease 2019 (COVID-19), produced by the SARS-CoV-2 virus, is an illness that can cause a severe acute respiratory syndrome and other multi-system life-threatening conditions in a relatively high proportion of infected individuals [1]. COVID-19 became a pandemic in spring 2020 and affected many countries that were unprepared to stop the spread of the virus that is readily transmitted from person to person predominantly through respiratory droplets [2]. To reduce the spread of SARS-CoV-2 during the first wave of COVID-19, governments of many countries declared home confinement measures, in addition to other isolation and hygiene measures and social (physical) distancing [3]. This entailed the suspension of most professional sporting activities for weeks, causing an unprecedented disruption of sport and training routines [4,5,6], potentially affecting players’ conditioning. In Spain, the lockdown enforced by health authorities lasted 8 weeks and entailed a strict quarantine that prohibited athletes from practicing any form of exercise outside of their own residence [6]. During the lockdown, the staff of Spanish professional football teams competing in *LaLiga* provided personalized training programs and/or organized video-based activities to reduce the potential loss of training-induced morphological and physiological adaptations [7]. Home training protocols were adapted to the conditions of players’ residences and considered that the execution of football-specific actions, such as accelerations/decelerations, changes of direction, and sprints, were difficult to replicate for most players under these conditions. 

Based on these limitations, some sports scientists suggested that the restrictions of home training and the absence of organized team training and competition would induce some detraining effects that may negatively impact athletes’ capacity to perform sport-specific actions, especially at high intensity [3,8,9], and that home confinement might increase the risk of injury when an athlete continues to compete [3,5]. Before resuming *LaLiga*, a field-based retraining period of 4 weeks was established and football teams used this period to optimize players’ physical condition, simulating a preseason phase. Additionally, football authorities allowed modifications in some in-game regulations (from three to five substitutions per match and additional refreshment pauses in each half of the match) [10]. A recovery period of at least 72 h was also set between matches to facilitate recovery due to the stress created by the congested calendar. A study by Brito de Souza et al. [11] showed that the running distance covered by players competing in *LaLiga* was similar after the lockdown to the period before the lockdown and to the last competitive phase of the previous season. However, there is no information to determine if this fixture congestion, as with previously reported scenarios, affected the injury incidence in Spanish professional football [12]. 

To the authors’ knowledge, only two studies [13,14] compared injury rates before and after the lockdown, both in the German football league (*Bundesliga*). In a study by Seshadari et al. [13], the authors reported a threefold increase in the incidence of injuries during football matches after the resumption of competition, especially with regard to muscle-type injuries. However, in a study by Krutsch et al. [14], the prevalence of injuries in matches played after the lockdown did not significantly differ from that in the pre-lockdown period, and they also found a significantly lower injury rate. The *Bundesliga* was the first football league to resume competition after just 10 weeks of suspension. It may have been the early resumption of the *Bundesliga* that contributed to the unusually high rate of injuries following the lockdown reported by Seshadri et al. [13]. In any case, both studies used injury data in the *Bundesliga* obtained from a public database where the definition of injury differs substantially from the previous consensus on football injury reporting [15], which may have affected the accuracy of the information collected. The aim of this investigation was to determine whether there is a difference in mean injury incidence in *LaLiga* players between the pre-lockdown period and post-lockdown period. For this study, we obtained data directly from the medical staff of teams competing in *LaLiga* that have access to players’ medical records, thus increasing the accuracy of the analysis. 

## 2. Materials and Methods

### 2.1. Participants

A total of 277professional football players (mean ± SD age: 27.7 ± 4.1 years; height: 180.3 ± 7.9 cm; body mass: 75.7 ± 6.4 kg) from 11 teams competing in *LaLiga* participated in this investigation. There were 96 defenders in the sample, 95 midfielders, 58 forwards, and 28 goalkeepers. Only players who remained in their teams throughout the season and who competed both before the suspension and after the resumption of the competition were included in the survey. The Institutional Board of the Miguel Hernandez University of Elche approved this study, which is in line with the latest version of the Declaration of Helsinki. All participants gave their consent to participate in the investigation, and injury and training/match load data were anonymized with an alphanumeric code applied to all participants.

### 2.2. Experimental Design

The current investigation is a comparative analysis of the injury incidence of professional football players before and after the suspension of *LaLiga* due to the COVID-19 pandemic. *LaLiga* 2019–2020 started on 16 August 2019, and each team participated in 25 football matches before being suspended on 12 March 2020. Football players were confined at home for 8 weeks (as of 14 March 2020), during which players could not exercise outside their homes. After that, the players started a phase of retraining in their clubs that lasted four weeks at their clubs according to the national COVID protocols. During the retraining period, professional teams prepared to return to the game following the recommendations of Spanish health and sports authorities, which established regulations allowing only individual exercise routines for the first week of retraining with progressive inclusion of small-group exercises until completing team trainings, and 11-per-side match simulation routines in the last weeks of the retraining period. The Position statement of the Royal Spanish Football Federation for the resumption of football activities after the COVID-19 pandemic, which contains a summary of the main recommendations for training and competition after the lockdown, can be consulted elsewhere [10].

The *LaLiga* competitions resumed on 11 June 2020, and the teams played the remaining 11 days of matches until 19 July 2020. Up to 5 substitutions were allowed during the rest of the competition and there was a mandatory refreshment break in each halftime of the match. For this study, we used data on injuries that the teams’ medical staff collected throughout the season as part of their medical tasks. The teams collected injury data using the same methods before and after the suspension of *LaLiga* and injury analysis was performed taking into account the injury date. To avoid the interference of the preseason and the retraining periods, only injuries sustained during the competition calendar (i.e., from 16 August 2019 to 9 March 2020 for the period before the suspension and from 11 June to 19 July 2020 for the period after the resumption of the competition) are included in the analysis. Initially, all 20 teams competing in *LaLiga* were invited to participate in the study, but only 11 teams agreed to participate, so only their data were analyzed.

### 2.3. Injury Data Collection

Injury data were obtained prospectively during the 2019–2020 competitive season. All injuries were diagnosed, classified, and recorded by the medical staff of the football teams using the classification system developed by the IOC [16]. Anonymized, recorded data were sent to the study group via an electronic database with no information that could reveal the identity of the player. For each injury, the region, body area, type of injury, affected tissue, examination findings and type of pathology were recorded, and diagnoses made were assisted by various additional medical examinations (e.g., X-ray, ultrasound, magnetic resonance imaging, etc.) depending on the type of injury. Injuries are divided according to the mode of occurrence into acute and recurrent/overuse injuries, and acute injuries are further divided according to the mechanism of occurrence into contact and noncontact injuries [16]. Training and competition injuries were also catalogued according to the IOC consensus statement, while injuries during warm-up and cool-down exercises were reported as training injuries [16]. As for recurrent injuries (i.e., an injury of the same type and at the same site), they were considered a new injury in cases where the player returned to full training and was available for competitions for at least one week. Finally, we used the definition of the IOC consensus statement to classify injury severity (i.e., number of days lost from normal training and competition), and then players were grouped into the following clusters: 0 days (no time loss), 1–7 days, 8–28 days, and more than 28 days [16]. The incidence of injuries was calculated in total and separately for match and training injuries, using match and training exposure data for each participant in this study. To determine the number of injuries per matchday, all injuries at training sessions held during the match preparation microcycle (including tapering and recovery sessions) and the injuries suffered during the match were included. Lastly, the rating of perceived exertion (RPE) was collected in a team to exemplify players’ perceived exercise before and during home confinement. 

### 2.4. Statistical Analysis

Statistical analyses were carried out using the software IBM SPSS Statistics for Macintosh, Version 26.0 (IBM Corp., Armonk, NY, USA). Initially, overall injury incidence was calculated independently for each team by using the number of injuries and match/training exposure data before the suspension and after the resumption of the competition. Afterwards, overall injury incidence in these two periods was compared with paired *t*-tests. The same procedure was performed for the comparison of injury incidence according to the tissue affected (i.e., muscle, ligament, bone, cartilage) or to compare match and training injury incidence between the two periods under investigation. Injury characteristics before the suspension and after the resumption of the competition were compared by using crosstabs and χ^2^ tests, including adjusted standardized residuals. For this analysis, injuries were classified according to each characteristic and frequencies were independently calculated for before the suspension and after the resumption of the competition. The significance level was set at *p* < 0.050.

## 3. Results

In the period before the suspension of the competition due to the COVID-19 pandemic (i.e., the first 27 matchdays), there were a total of 249 injuries recorded in the teams under investigation. This represented an overall injury incidence of 4.2 injuries per 1000 h of exposure (range = 1.9–7.7 injuries per 1000 h of exposure). After the resumption of the competition, there were a total of 69 injuries and the injury incidence was similar to before the competition suspension (5.4; 1.5–9.6 injuries per 1000 h of exposure, *p* = 0.338). Injury incidence before suspension and after the resumption of the competition was similar for muscle/tendon, ligament/joint/capsule, bone, and cartilage/synovium/bursa injuries (Table 1). Match injury incidence in matches was 11.4-fold higher than training injury incidence but the resumption of the competition did not modify match/training injury incidence (Table 1). Injury incidence before and after lockdown was similar for goalkeepers (from 3.3 to 1.3 injuries per 1000 h of exposure), wing-back players (from 4.8 to 6.7 injuries per 1000 h of exposure), center-back players (from 4.3 to 5.1 injuries per 1000 h of exposure), center-midfielders (from 6.4 to 6.6 injuries per 1000 h of exposure), winger players (from 1.7 to 2.4 injuries per 1000 h of exposure), and center-forward players (from 5.0 to 10.8 injuries per 1000 h of exposure; all *p* > 0.05). 

**Figure 1:**
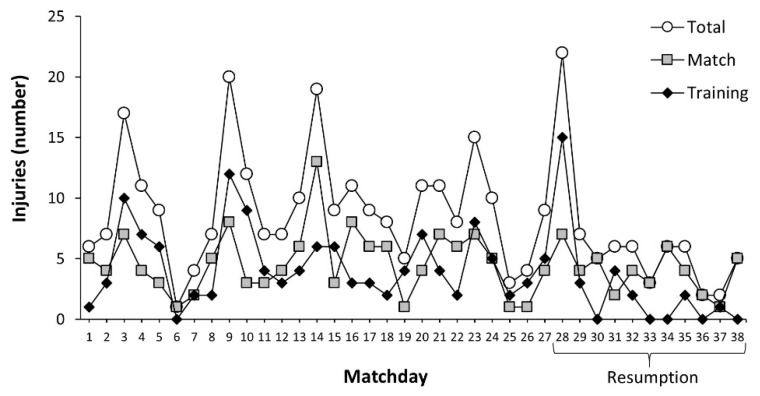
Number of injuries per matchday in professional football players before the suspension of LaLiga due to the COVID-19 pandemic and after the resumption of the competition.

Figure 1 contains the number of injuries sustained during match and training in each of the matchdays that composed the season. Matchday 28 (first match after suspension) was the fixture with the highest number of injuries (22 injuries, 15 training injuries and 7 match injuries), although a similar value was obtained on matchday 9 (20 injuries, 12 training injuries and 8 match injuries). Additionally, the number of injuries per matchday in the fixtures performed after the resumption of the competition was similar to that before the suspension (Figure 1).

Table 2 contains the distribution of injuries before suspension and after the resumption of the competition according to tissue affected, body location, mode of onset, presence of contact, condition, recurrency, severity, and position in the field of the player. 

**Figure 2:**
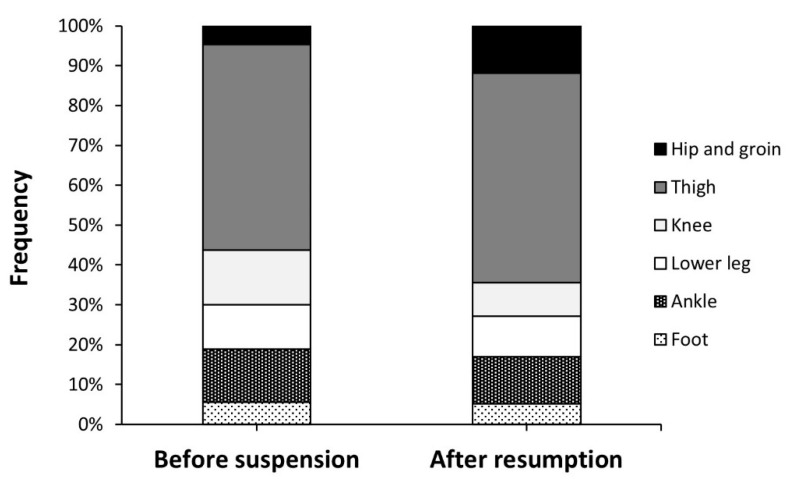
Distribution of lower limb injuries in professional football players before the suspension of *LaLiga* due to the COVID-19 pandemic and after the resumption of the competition.

Muscle/tendon was the most commonly injured tissue before suspension and after the resumption of competition, and there was not any difference in the distribution of injuries according to tissue affected. The distribution of injuries according to body location was not affected by the suspension of the championship, with lower limb being the most common in both scenarios. Within the lower limb, the distribution of injuries was also not affected by the suspension of the competition (Figure 2; *p* = 0.398). 

**Figure 3:**
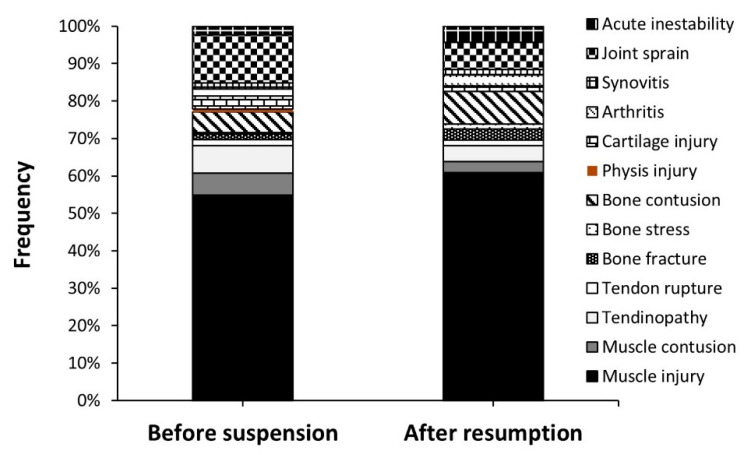
Distribution of injuries according to their type in professional football players before the suspension of *LaLiga* due to the COVID-19 pandemic and after the resumption of the competition.

In the resumption of the competition, there was no change in the distribution of injuries according to their mode of onset, presence of contact, or condition, and it did not affect the distribution of injuries among playing positions (Table 2). However, there was a tendency for a higher frequency of injuries that required only 1–7 days to return to play after the resumption of the competition, while the proportion of severe injuries (i.e., more than 28 days of recovery) was reduced. Finally, the distribution of injuries according to their type was not affected by the resumption of the competition (Figure 3; *p* = 0.766). 

**Figure 4:**
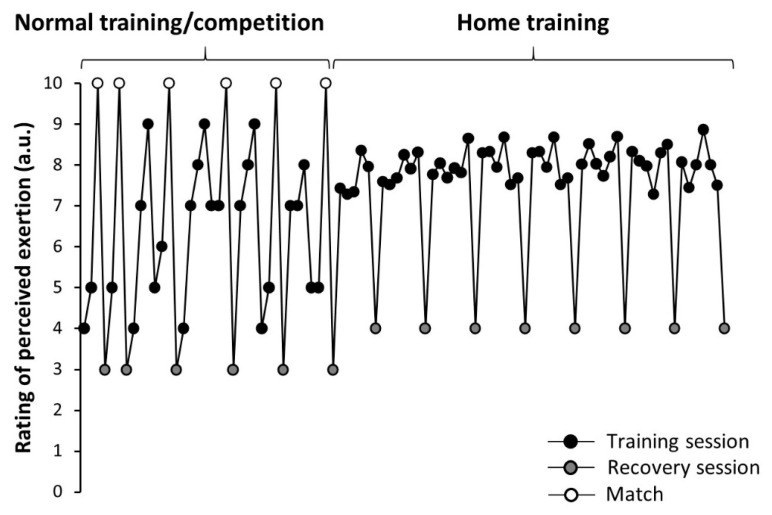
Example for the rating of perceived exertion obtained at the end of each training session in one team of *LaLiga* during 7 weeks prior to lockdown due the COVID-19 pandemic (normal training and competition) and during the 8 weeks that lasted during home confinement (home training).

Figure 4 depicts RPE values obtained at the end of each training session in one team of *LaLiga* during 7 weeks prior to lockdown and for the 8 weeks that lasted during home confinement (home training). Each dot represents the mean value for all the players who took part in the training session/match. During the normal training/competition period, the team played one match per week, except for the first week. As is shown in Figure 4, players perceived exertion was variable during the period of normal training and competition, with values of 10 a.u. of RPE in the days with a match and values of 3 a.u. of RPE in the day after the match that corresponded to a recovery session. On the other hand, RPE values were more stable during home confinement (between 7 and 8 a.u.), as the load was similar for each day and there was no need to reduce training load to prepare for a match. However, the strength and conditioning staff of this team prescribed a training day per week during home confinement with a lower load (including light aerobic exercise and stretching routines), and the RPE value for these days was 4 a.u. 

## 4. Discussion

The aim of this study was to compare injury incidence and epidemiology in *LaLiga* before suspension and after the resumption of the competition due to the COVID-19 pandemic. The events that led to this research happened during the 2019–2020 season and implied the suspension of competition for 12 weeks, including 8 weeks of home isolation and 4 weeks of retraining. After this period, the competition was resumed to complete the 11 fixtures remaining. The rationale for this investigation was based on previous reports suggesting that, due to the insufficient stimuli and the absence of organized training and competition during home isolation, football players were likely exposed to some level of detraining that could increase the risk of injury after the lockdown [3,15]. However, health and football authorities enforced a set of regulations for the resumption of the competition and developed strategies for injury prevention [10]. The results of this study indicate that the injury rate in *LaLiga* was not affected by lockdown, at least in comparison to the values of the same season before the suspension of the championship. The analysis presented in this investigation suggests that the conditions set to resume *LaLiga* probably aided in producing an unchanged injury pattern after the lockdown. 

The home-based training programs during home isolation were individually designed by the strength and conditioning staff of the clubs to optimize each player’s conditions. Overall, the objective of home-based training was to induce a training load similar to normal training. For this reason, training sessions were designed to produce exercise exposure times and rating of perceived exertions aligned with players’ conventional in-season training. However, during home confinement, their load was more consistent because players did not compete, and it was unfeasible to reproduce the demands of competition with home training exercises (Figure 4). Although there were some differences in the exercise programs designed by each football team, most teams employed six training sessions per week with a recovery/free session. Treadmill running and indoor cycling were the most common exercise activities used to maintain endurance performance. Additionally, strength training was incorporated into each program and both body loads, and external weights were utilized, depending on the equipment available for each player. The staff of the teams used video calls with the players to supervise the exercise routines and to provide feedback and motivation, especially as the duration of the confinement was longer than initially planned. The training program was accompanied by diet supervision in most football clubs to reduce potential deviation in players’ body mass and body composition during the lockdown. 

The only published information describing the effect of lockdown due to the COVID-19 pandemic on injury epidemiology has been provided with data from the *Bundesliga* [13,14]. Seshadari et al. [13] found a significant increase in the number of injuries per match in the *Bundesliga* after the resumption of the competition, at least in comparison to pre-lockdown. This increase in the rating of match injury was mostly associated with muscle-type injuries, particularly because of an abnormal number of injuries at the first matchday after the resumption of the championship. However, in the study by Krutsch et al. [14], injury incidence in the matches played after the lockdown were similar to the pre-lockdown period. Interestingly, these two investigations present some contradictory results despite analyzing the same event. The lack of agreement between investigations may be due to differences in data collection, as both Seshadari et al. [13] and Krutsch et al. [14] obtained injury data from public databases instead of gathering information directly from the football clubs. Specifically, Seshadri et al. [13] used a Transfermarkt to report and quantify injury rates, while Krutsch et al. [14] used information from the German kicker^®^ sports magazine. The obtaining of injury data from a public database could produce some errors in the quality of the data obtained because the information is not directly from the clubs. Additionally, in the study by Seshadri et al. [13], only the injury rate per match was calculated, as this is the only exposure publicly available, while the current analysis presents information of both match and training exposures. If we assume that the methods for obtaining injury data are comparable among investigations, a third explanation for the differences in the studies’ outcomes is associated with how *LaLiga* and *Bundesliga* resumed and organized the competition to finish their respective championships. The *Bundesliga* returned on 16 May of 2020 after 10 weeks of suspension and it was the first professional sporting organization to restart the competition after the first wave of COVID-19. *LaLiga* resumed competition on 8 June of 2020, because a retraining period of 4 weeks was established. In both cases, *LaLiga* and *Bundesliga* teams prepared individual training programs to be followed by their players at home during the lockdown, adapted to their conditions for training for each player. Hence, it is probable that the early resumption of the *Bundesliga* may have affected the high injury incidence reported in this competition during the first matchdays. Significantly, the time between matches in the *Bundesliga* to finish the nine matchdays left to complete the championship (one match every 4.6 days) was even longer than the time between matches in *LaLiga* (one match every 3.5 days) as there were 11 matchdays left to conclude the Spanish national league [11]. A final explanation is that the injury rate in the *Bundesliga* was unaffected after the lockdown, as suggested by the study by Krutsch et al. [14]. In any case, while what happened in the last matches after the lockdown in the *Bundesliga* is not completely clear due to discrepancies in studies and the obtaining of injury data from public databases, we have a clear picture in *LaLiga* that indicates that injury incidence was similar after the lockdown, as we have obtained data directly from the clubs, including match and training exposure data.

In accordance with previous systematic reviews and meta-analyses carried out in professional football teams [17,18], most of the reported injuries in the present study were muscle-type, were located in the lower limb (specifically in the thigh), and occurred acutely. In addition, similarly to previous findings [17,18], the present study showed a higher injury incidence during matches than during training. However, the current investigation is innovative because this injury pattern was similar in the 27 matchdays performed before the suspension of the competition than in the 11 matches carried out after the resumption. This finding contrasts with the suggestion of previous authors who anticipated a higher injury risk/incidence, especially for muscle-type injury, after the resumption of competition due to the combination of a congested calendar and the potential detraining effects induced by home confinement [5]. However, as expressed above, football teams encouraged their players to keep training routines at home and specifically prepared players for the retraining period before the resumption of the competition as a preseason. Additionally, the adaptations of in-game regulations likely helped to counteract the deleterious effects of the congested calendar. Lastly, it is likely that the pattern of injury after the lockdown was similar to before the lockdown because the demands of the game, in terms of running distances, were also maintained, as recently shown by Brito de Souza et al. [11]. In that study, running parameters in the last 11 fixtures of *LaLiga* 2019–2020 (matchdays 28 to 38) were compared to the period prior to the suspension (i.e., matchdays 1 to 27) and to the same last 11 fixtures of the previous season (2018–2019). Such analysis allowed a pairwise comparison of the fixtures after the resumption of the competition to the same matchday of a normal season because previous data indicate that running performance is lower in the first fixtures of a season [6], limiting the applicability of comparing running data before the suspension and after the resumption of the championship. The analysis by Brito de Souza et al. [11] showed that *LaLiga* teams maintained high-intensity running (i.e., <21 km/h) and the number of sprints after the resumption of competition, and this was evident when comparing data from the “COVID-19 season” to the “control” 2018–2019 season. In fact, in the fixtures after the resumption of the competition, there was an increase in the total running distance and the distance covered at low running velocity. It was argued by the authors of that study that the increases in total running distance and in low-intensity running were likely facilitated by the longer match duration due to the refreshment pauses and the possibility of reaching up to five substitutions per match. All this information suggests a scenario where the in-game regulations introduced to complete the championship after the lockdown in the 2019–2020 season aided in maintaining high-intensity running during matches while reducing the overall demands of the game due to longer matches and a higher number of substitutions. 

While the results of this study provide information regarding the lack of negative effects of the lockdown on injury epidemiology in the matchdays played in *LaLiga* 2019–2020 after the resumption of the championship, some limitations to the study must be acknowledged. Firstly, the present study has been performed in a specific sample of professional football players competing in the top division of Spanish football and these findings may not be applicable to other national football leagues. The differences in the duration of confinement and in the post-lockdown calendar suggest that an individual study should be carried out for each national football league. Second, we were able to obtain reliable and valid injury data from professional football teams, which represents 55% of the total number of teams competing in the first division of *LaLiga*. Still, it is probable that the remaining nine teams presented injury incidences and injury patterns after the resumption of the competition different to the ones presented in this investigation. Third, we compared injury rates before and after the suspension/resumption of the competition to assure that the teams maintained the sample players and team coaches. However, this implies the comparison of different moments of the season, which may have affected some of the results of this investigation. Finally, in the period before the suspension of *LaLiga,* some of the teams were also competing in international competitions, such as UEFA Champions League, UEFA Europa League, or in other national competitions, such as *Copa del Rey*. Additionally, some of the players competed with their national teams in this pre-lockdown period. Hence, although there were 27 matchdays of *LaLiga* played in 30 weeks until the competition was suspended due to COVID-19, the real number of matches per week for some teams/players could have been similar to the period after the lockdown.

## 5. Conclusions

In summary, the current data showed that injury incidence, distribution, and description (tissue affected, body location, and condition) in professional football players from 11 different football teams belonging to the top division of the Spanish football league (*LaLiga*) did not differ in the games played after suspension of the championship due to the first wave of COVID-19. This suggests that the specific conditioning strategy employed for the clubs to maintain the physical fitness of their players during the lockdown, in addition to the modified conditions established for the resumption of *LaLiga* (i.e., 4-week retraining period, 72 h between matches, up to five substitutions, and in-game refreshment pauses), likely aided to prevent an unusually high injury incidence in the matchdays played after the lockdown.

## Figures and Tables

**Table 1:** Incidence of injuries shown according to tissue damage and time of injury (in training or during the match) in professional football players for the matchdays played before the suspension of *LaLiga* due to the COVID-19 pandemic and after the resumption of the competition.

Injury Incidence (Injury/1000 h of Exposure)	Before Suspension	After Resumption	*p* Value
Muscle/tendon	2.6 (1.2–3.5)	3.4 (0.9–7.6)	0.152
Ligament/joint/capsule	0.8 (0.2–2.3)	0.4 (0.0–1.2)	0.062
Bone	0.4 (0.0–0.9)	0.7 (0.0–1.6)	0.318
Cartilage/synovium/bursa	0.4 (0.0–1.5)	0.7 (0.0–2.8)	0.305
Training	2.3 (0.9–4.6)	2.5 (0.0–4.0)	0.219
Competition	25.9 (11.2–38.2)	21.5 (5.5–49.6)	0.679

Data represent the mean (range) for injury incidence of 11 professional football teams.

**Table 2:** Conditions of injury in professional football players for the matchdays performed before the suspension of *LaLiga* due to the COVID-19 pandemic and after the resumption of the competition.

Characteristic	Injury Condition	Before Suspension	After Resumption	*p* Value
Tissue affected	Muscle/tendon	156 (62.7)	44 (63.8)	0.345
	Ligament/joint/capsule	45 (18.1)	7 (10.2)	
	Bone	23 (9.2)	9 (13.0)	
	Cartilage/synovium/bursa	25 (10.0)	9 (13.0)	
Body location	Head	5 (2.0)	2 (2.9)	0.948
	Upper limb	8 (3.2)	2 (2.9)	
	Trunk	15 (6.0)	5 (7.2)	
	Lower limb	221 (88.8)	60 (87.0)	
Mode of onset	Acute sudden	161 (64.7)	40 (58.0)	0.614
	Repetitive gradual	83 (33.3)	27 (39.1)	
	Repetitive sudden	5 (2.0)	2 (2.9)	
Contact	Direct contact	55 (22.1)	13 (18.8)	0.724
	Indirect contact	10 (4.0)	2 (2.9)	
	No contact	184 (73.9)	54 (78.3)	
Condition	Training	123 (49.4)	27 (39.1)	0.115
	Competition	126 (50.6)	44 (60.9)	
Recurrency	New	229 (92.0)	64 (92.8)	0.830
	Recurrent	20 (8.0)	5 (7.2)	
Severity	0 days	2 (0.8)	1 (1.4)	0.065
	1–7 days	80 (32.1)	32 (46.4)	
	8–28 days	113 (45.4)	29 (42.0)	
	>28 days	54 (21.7)	7 (10.1)	
Position	Goalkeeper	23 (9.2)	2 (2.9)	0.295
	Wing-back	56 (22.5)	17 (24.6)	
	Center-back	51 (20.5)	13 (18.8)	
	Center-midfielder	64 (25.7)	15 (21.7)	
	Winger	20 (8.0)	6 (8.7)	
	Center-forward	35 (14.1)	16 (23.2)	

Data represent the number (frequency) for 249 injuries recorded before suspension and 69 injuries recorded after resumption of the championship in 11 professional football teams.

## Data Availability

The data presented in this study are available on request from the corresponding author. The data are not publicly available due to restrictions of the club where the data were obtained.
